# Morphologies in‐between: The impact of the first steps on the human talus

**DOI:** 10.1002/ar.25010

**Published:** 2022-06-21

**Authors:** Carla Figus, Nicholas B. Stephens, Rita Sorrentino, Eugenio Bortolini, Simona Arrighi, Owen A. Higgins, Federico Lugli, Giulia Marciani, Gregorio Oxilia, Matteo Romandini, Sara Silvestrini, Fabio Baruffaldi, Maria Giovanna Belcastro, Federico Bernardini, Anna Festa, Tamás Hajdu, Orsolya Mateovics‐László, Ildiko Pap, Tamás Szeniczey, Claudio Tuniz, Timothy M. Ryan, Stefano Benazzi

**Affiliations:** ^1^ Department of Cultural Heritage University of Bologna Ravenna Italy; ^2^ Department of Anthropology Pennsylvania State University State College Pennsylvania USA; ^3^ Department of Biological, Geological and Environmental Sciences – Bigea University of Bologna Bologna Italy; ^4^ Human Ecology and Archaeology (HUMANE) IMF, CSI0C Barcelona Spain; ^5^ Research Unit Prehistory and Anthropology, Department of Physical Sciences, Earth and Environment University of Siena Siena Italy; ^6^ Laboratory of Medical Technology IRCCS Istituto Ortopedico Rizzoli Bologna Italy; ^7^ Department of Humanistic Studies Università Ca'Foscari Venezia Italy; ^8^ Multidisciplinary Laboratory Abdus Salam International Centre for Theoretical Physics Trieste Italy; ^9^ Department of Biological Anthropology, Institute of Biology, Faculty of Science Eötvös Loránd University Budapest Hungary; ^10^ Archaeological Heritage Directorate, Hungarian National Museum Budapest Hungary; ^11^ Department of Biological Anthropology, Institute of Biology, Faculty of Science and Informatics Szeged University Szeged Hungary; ^12^ Department of Anthropology Hungarian Natural History Museum Budapest Hungary; ^13^ Centre for Archaeological Science University of Wollongong Wollongong New South Wales Australia; ^14^ Department of Human Evolution Max Planck Institute for Evolutionary Anthropology Leipzig Germany

**Keywords:** bipedalism, geometric morphometric, human growth, ontogeny, trabecular analysis

## Abstract

**Objective:**

The development of bipedalism is a very complex activity that contributes to shaping the anatomy of the foot. The talus, which starts ossifying in utero, may account for the developing stages from the late gestational phase onwards. Here, we explore the early development of the talus in both its internal and external morphology to broaden the knowledge of the anatomical changes that occur during early development.

**Materials and Methods:**

The sample consists of high‐resolution microCT scans of 28 modern juvenile tali (from 36 prenatal weeks to 2 years), from a broad chronological range from the Late Roman period to the 20th century. We applied geometric morphometric and whole‐bone trabecular analysis to investigate the early talar morphological changes.

**Results:**

In the youngest group (<6 postnatal months), the immature external shell is accompanied by an isotropic internal structure, with thin and densely packed trabeculae. After the initial attempts of locomotion, bone volume fraction decreases, while anisotropy and trabecular thickness increase. These internal changes correspond to the maturation of the external shell, which is now more defined and shows the development of the articular surfaces.

**Discussion:**

The internal and external morphology of the human talus reflects the diverse load on the foot during the initial phases of the bipedal locomotion, with the youngest group potentially reflecting the lack of readiness of the human talus to bear forces and perform bipedal walking. These results highlight the link between mechanical loading and bone development in the human talus during the acquisition of bipedalism, providing new insight into the early phases of talar development.

## 1 INTRODUCTION

During the walking cycle, the human foot is the only element that makes contact with the ground, accommodating its irregularities while maintaining balance. Moreover, it supports the body weight, while also serving both as a shock‐absorber and a lever for the transmission of propulsive forces (Hallemans et al., 2006). The adult human foot is well adapted and highly specialized to perfectly perform all these tasks. Although the origin of human locomotion is still debated, numerous studies have contributed to deepening our knowledge of the evolution of bipedalism (Almécija et al., 2013; Desilva, Carlson, et al., 2018; DeSilva, Gill, et al., 2018; Finestone et al., 2018; Frelat et al., 2017; Harcourt‐Smith, 2010; Holowka et al., 2017a, 2017b; Marchi, 2015; O'Neill et al., 2015; Ryan & Shaw, 2012; Ryan & Van Rietbergen, 2005; Saers et al., 2016, 2020; Shaw & Ryan, 2012; Sorrentino, Belcastro, et al., 2020; Sorrentino, Stephens, et al., 2020; Swan et al., 2020; Ward, 2002). The hominid foot has been largely investigated to comprehend how its anatomy reflects adaptations to locomotory behaviors, levels of mobility, and substrate preferences (DeSilva et al., 2019; Fernández et al., 2018; Harper et al., 2021; Holowka & Lieberman, 2018; Prang, 2015; Sorrentino, Carlson, et al., 2020; Sorrentino, Stephens, et al., 2020; Su & Carlson, 2017; Turley et al., 2015). Nevertheless, the development of human locomotion is less investigated, and the morphological changes linked to the acquisition of bipedalism are still to be thoroughly understood.

Human locomotion has a really long and complex acquisition path. In the immediate postnatal period, newborns are fully dependent on their caregivers. Consequently, the development of locomotory skills is strictly correlated with the socio‐cultural background, the progress of social abilities, and cultural behaviors. Besides, the maturation of different systems, such as the musculoskeletal and neurological ones, also has its influence. Moreover, neonates exhibit some reflexes, which are part of the “transitory, primitive behavior” (Lacquaniti et al., 2012) that are fundamental for their basic and vital actions, for example, sucking reflexes for feeding. These reflexes might also be observed in the limbs. In fact, the fetus moves and kicks, with an activity peak around the 14th–16th week. Movements become more constrained as the pregnancy approaches the end due to a lack of space (Adolph & Franchak, 2017), though neonates might replicate many of these fetal movements, that is, stepping reflexes. During the first postnatal months, neonates struggle with gravity and the increase in leg fat. These two factors make their lower limbs more difficult to move and lift, but they soon learn how to cope with gravity, while muscles become stronger (Adolph & Franchak, 2017).

Due to the complexity of the background behind the conquest of bipedalism, locomotory behaviors should not be interpreted without considering the toddler's broad context. Developmental milestones differ from infant to infant as well as from population to population. Different childrearing practices may affect the timing of locomotor development (Adolph & Franchak, 2017), and skills may be acquired in an altered order as infants can skip or revert stages. Therefore, developmental paths are far from linear. Unfortunately, the most common charts for the evaluation of children's development reflect the researchers' cultural bias and samples (Adolph & Franchak, 2017). At first, researchers tried to standardize the motor progresses based on samples from the US middle‐class of European descent (Karasik et al., 2015). Consequently, they observed a rather homogenous sample, with no or scarce cultural variation. Based on these observations, infants from other cultures have been defined as precocious or delayed (Lerner et al., 2015).

Nevertheless, researchers may observe a rather defined pattern of progression, which begins in the upper part of the body (e.g., head, trunk) and moves to the lower (e.g., hips, lower limbs). Briefly, infants may usually sit independently, without the support of their hands at around six postnatal months. Considerable cross‐cultural differences have been described in the onset of sitting. Variability depends on childrearing practices, but it is also linked to differences in flooring and/or furniture (Karasik et al., 2015). Subsequently, some infants may begin to crawl at around 9 months. Usually, infants first start moving forward on their abdomen; then, they try to support the upper body with their arms and, when they are strong enough, to propel the upper body. Finally, they learn to use their hands and knees to move forward. The motor phases are not independent events; they are strictly correlated. They should be interpreted as part of a unique event, and the observation of the progress should take into account that infants may smoothly transition from crawling to sitting (Soska et al., 2015), and vice versa, at around 7–8 months (Adolph et al., 2018). If crawling is performed, it may facilitate the development of the sensory and motor system, memory, and spatial cognition (Xiong et al., 2018).

The average age of the onset of bipedal locomotion is around 12 months (Karasik et al., 2012; Sutherland et al., 1980), though the onset has a much wider range (8–18 months) and depends on numerous variables (Adolph & Franchak, 2017). If performed, the transition from crawling to walking might change depending on various factors, such as the transportation of objects or interactions with people (Karasik et al., 2015). Independent walking starts between 10 and 17 months (Zeininger et al., 2018). A daily walking practice may lead infants to start walking slightly earlier than their peers who do not practice constantly. Experience is a fundamental factor that plays a key role in individual differences (Adolph & Robinson, 2013). In addition, in some Caribbean and African cultures, where parents make the children practice or give them massages as part of a daily care routine, infants walk sooner than others from similar cultural backgrounds who do not receive the same care routine (Adolph & Franchak, 2017). Toddlers must be strong enough to support the weight of their body while efficiently pushing themselves forward and coping with the challenges of imbalance. They usually keep their arms high as a further aid to improve balance (Karasik et al., 2012). In general, new walkers have a wide walking base characterized by a small step length and slow pace. Contact with the floor is made with a flat foot and an extended knee. The centers of pressure are more evenly distributed than in adults, due to the lack of a proper heel‐strike and toe‐off pattern, as well as an immature longitudinal arch. A more efficient heel‐strike develops at around 2 years of age (Zeininger, 2013).

Along with the progress in motor skills, the morphology of the foot changes. The toddler's foot consists mostly of ossific nuclei and cartilage, connected by soft tissues. At about 12 months, the talus, the calcaneus, the cuboid, the metatarsals, and the phalanges contain their primary ossification centers, still surrounded by cartilaginous tissue (Hallemans et al., 2003). The ossification of the talus starts around the sixth‐seventh prenatal month, usually from one center, even though more than one nucleus has been noted (Gray et al., 1957). Therefore, the talus is, ideally, a good “recorder,” as it may account for all the developing stages, starting from the late gestational phase. The perinatal talus has an ellipsoid form, with two main indentations on the plantar and dorsal surfaces that mark the position of the future sulcus tali and neck, respectively, separating the future articular facets. In the neonatal talus, the angle formed by the neck and head is reported to be around 130°–140°, which increases during growth to reach adult proportions (150°) (Cunningham et al., 2016). According to Scheuer and Black (2004), the reduced angle is linked to the inverted neonatal foot, whereas the increase in angle has been associated with the onset of bipedal gait, at about 10–12 months.

Studies on the ontogenetic changes linked to the modifications in the load‐bearing forces that act on the foot are still scarce (Saers et al., 2020; Zeininger et al., 2018). To the knowledge of the authors, there are few studies on the developing talus, which do not take into account the earlier phases of talar growth (Hellier & Jeffery, 2006; on the development of the talocrural joint: Turley & Frost, 2014; Turley et al., 2018). Since the foot is a highly derived and functionally significant region for investigating ontogenetic changes linked to the development of bipedal locomotion, we will focus on the study of talar growth in humans in an attempt to add information about the development of this important bone.

### 1.1 Shape and function

The loading of the skeleton is crucial to reach and maintain an adequate and functional bone mass. This process is ascribable to highly regulated mechanisms, that allow bone modeling through site‐specific activation of osteoblasts and osteoclasts (Pivonka et al., 2018). During the first 2 years of life, the bone modeling rate is higher than in adults, with estimates of neonatal modeling at 50% per annum, compared to just 5% per annum in adult life (Walker, 1991). Movements start in utero and continue during postnatal life. Previous studies have shed light on the type, intensity, and duration of movements during different gestational periods (DiPietro et al., 2010; Einspieler et al., 2008). It is also well known that the intrauterine environment is drastically different from the extrauterine one, the former being a protected space with virtually no gravity and no loads since the fetus lives immersed in the amniotic fluid. Nonetheless, fetal movements generate forces that are fundamental in the process of growth, aiding in shaping the bones into their adult morphology (Carter & Beaupre, 2001). Mechanical stimuli in utero and during early postnatal life are vital for normal bone development, and may serve to reinforce both bone shape and structure (Cunningham & Black, 2009a, 2009b). The external shape of bones clearly reflects a genetic blueprint and phylogenetic history. This makes it even more challenging when trying to discern the plastic and functional characteristics from the programmed ones.

The internal bone structure may help to provide a more complete picture. Initial growth of cancellous bone is likely driven largely by programmed developmental patterns. The responsiveness of cancellous bone to the mechanical environment makes it a potentially powerful tool for understanding the loading and functional environment of a bone (Kivell, 2016). In fact, adult trabecular bone structure is the result of the loading history that affects bone growth during ontogeny (Gosman & Ketcham, 2009; Ryan & Krovitz, 2006). During development, bone reacts to external stimuli by both changing the dimensions and orientation of articular facets (Hellier & Jeffery, 2006) and by adapting the trabecular struts through modeling in response to the changes in load‐bearing (Cunningham & Black, 2009b). Skedros et al. (2007) showed that adult skeletal structure is derived from a combination of both genetic and epigenetic factors. To sum up, ontogenetic studies of both internal and external structures are of paramount importance for deepening our understanding of the processes that lead to adult morphology (Kivell, 2016; Saers et al., 2020).

Aware of the fact that the fetal talus is not yet fully ossified at birth, and because motor skills appear to develop rapidly in the first 3 to 6 months following the onset of independent locomotion before slowing down (Adolph et al., 2003), we aim to study the morphological changes of the talus from approximately 36–38 prenatal weeks (i.e., from the ossific nucleus) to 2 years of age. The goals of this study are twofold: (1) we explore the global talar growth, describing the changes that occur in the human talus from the last prenatal weeks to 2 years of age, in both the internal and external morphology to broaden the knowledge on the anatomical changes during early development; (2) we quantify and compare the morphological differences that may exist in the talus before the onset of significant mechanical loading (circa 6 months) and after the onset of locomotor loading, as children start sitting, moving, and exploring the space around them. Here, we characterize and analyze external shape change along with trabecular bone structural changes in the human talus during growth in relation to the acquisition of bipedal walking. Based on previous literature (Chevalier et al., 2021; Colombo et al., 2019; Hellier & Jeffery, 2006; Raichlen et al., 2015; Ryan et al., 2017; Saers et al., 2020; Turley & Frost, 2014; Zeininger et al., 2018), we expect to see differences in both the external and internal talar morphologies after about 6 months of age, when the foot starts to be loaded more frequently and consistently with locomotor movements. We expect the external shape to start modifying its morphology, with an initial development and expansion of the neck area and head, as a preparation for the initial and imminent loading. After around six postnatal months, we expect to see a more mature talar shape, with noticeable morphological changes, especially in the trochlea, head, and neck. These areas are pivotal for the transmission of the forces generated by the initial loading of the foot. We do not expect to see a great development of the posterior calcaneal facet due to the absence of a proper heel‐strike motion during this timeframe (Zeininger et al., 2018). Considering the prenatal bone overproduction, which may serve as a calcium supply soon after birth (Acquaah et al., 2015), we expect to see a dense trabecular architecture in the youngest individuals, with numerous and thin trabeculae, closely packed together in a relatively isotropic structure (Saers et al., 2020). Trabecular bone is then resorbed during the first year of life, causing a decrease in bone volume fraction and trabecular number (Ryan et al., 2017; Saers et al., 2020). Moreover, the trabecular architecture is expected to change, as the foot begins to be loaded. Consequently, the trabecular structure of the post‐loading individuals is predicted to become more anisotropic and less dense, with fewer and thicker trabeculae.

## 2 MATERIAL AND METHODS

### 2.1 Sample

This study analyzes the early development of the talus in a sample consisting of 28 modern juvenile tali aged between 36 prenatal weeks and 2 postnatal years (Table 1). The sample is mixed, from a number of different sites and locations. Four individuals, with known age at death, sex, and cause of death (Belcastro et al., 2017), belonged to the Identified Human Skeletal Collection of the Certosa Cemetery (Bologna, Italy, 19th–20th Century). All the children in this collection died from acute diseases. The absence of chronic pathologies that might have lasted in time leads us to believe that this cohort did not suffer from prolonged periods of locomotor difficulties, which could have affected the development of locomotion. The archeological sample was selected from different sites, spanning from the Roman Imperial period to the modern age. Seven individuals came from the site of Norris Farms #36 (Illinois, USA), housed at the Illinois State Museum, for which the age at death was estimated based on tooth crown formation and dental eruption stages (Milner & Smith, 1990). They were part of the Oneota culture, dating to 1,300 AD (Milner & Smith, 1990). Sixteen individuals came from the Imperial Roman site of Velia. The site, originally founded by Greeks in 540 BC (Morel, 2006), is located on the Italian west coast, near Salerno (Campania, Italy). Numerous archeological campaigns have been held and, during the 2003–2006 campaigns (Fiammenghi, 2003), a necropolis with over 330 burials was discovered. This necropolis, which dated back to the I and II centuries AD, yielded both cremations and inhumations, with numerous juvenile burials. Age at death was estimated based on dental and skeletal maturation.

**TABLE 1:** Archeological sample

Site (location)	Specimen	Period	Age at death	Class	Cause of death	External morphological analysis	Trabecular analysis
Bologna (Italia)	58_M	XX c.	11 months	Post‐loading	Bronchitis		✓
	60_F		11 months	Post‐loading	Acute meningitis (brain fever)		✓
	14_M		1 year 5 months	Post‐loading	Chronic enteritis	✓	✓
	14_F		1 year 9 months	Post‐loading	Enteritis	✓	✓
Norris Farm (Illinois, USA)	821045	1,300 AD	3 months	Pre‐loading			✓
	821369		8 weeks	Pre‐loading			✓
	820614		12 months	Post‐loading			✓
	821051		7.5 months	Post‐loading		✓	✓
	821046		1.5 years	Post‐loading		✓	✓
	821026		2 year	Post‐loading		✓	✓
	821207		2 years	Post‐loading		✓	✓
Perkáta‐Nyúli dűlő (Hungary)	516	XIV–XVI c.	1.5–3 years	Post‐loading		✓	✓
	655		1–3 years	Post‐loading		✓	✓
Velia (Italy)	T344us1566	Roman (Imperial Age, I‐II c. AD)	Perinate	Pre‐loading		✓	✓
	T417us2355		Perinate	Pre‐loading			✓
	T350us1594		Perinate	Pre‐loading		✓	✓
	T322us1436		Perinate (36–39 weeks)	Pre‐loading			✓
	T315us1398		Perinate (36–40 weeks)	Pre‐loading		✓	✓
	T383us2175		Perinate (38 weeks ca)	Pre‐loading		✓	✓
	T305us1344		0–3 months	Pre‐loading		✓	✓
	T300us1167		0–6 months	Pre‐loading		✓	✓
	T441us2538		0–6 months	Pre‐loading			✓
	T368us2069		9–12 months	Post‐loading		✓	✓
	T398us2239		6–8 months	Post‐loading		✓	
	T289us1098		9–12 months	Post‐loading			✓
	T442us2545		6–9 months	Post‐loading			✓
	T286us1071		1.5–2 years	Post‐loading			✓
	T434us2454		1–1.5 years	Post‐loading		✓	✓
	T415us2344		1–1.5 years	Post‐loading		✓	✓
						18	28

*Note*: Individuals are listed by population.

Two individuals came from Perkáta‐Nyúli dűlő (Hungary) and are stored at the Archeological Heritage Directorate of the Hungarian National Museum in Budapest. Perkáta‐Nyúli dűlő, in the Transdanubian region of Hungary, is a Cuman cemetery that counts more than 4,000 graves, some of which are from the transition period of the Cuman integration (Hatházi, 2004). The site was found during motorway construction works between 2009 and 2010 by the Field Service for Cultural Heritage. Age‐at‐death was estimated based on the development of deciduous and permanent teeth (Moorrees et al., 1963a, 1963b; Smith, 1991). When no teeth were available, diaphyseal length (Stloukal & Hanáková, 1978) and epiphyseal fusion (Ferembach, 1980) were used for age estimation (László, 2018; Szeniczey et al., 2019).

All the specimens were selected based on their good condition, with minimal or no damage, and no signs of pathology. The left side was preferred; when missing or incomplete, the right one was selected and mirrored. To quantify morphological and structural differences in relation to changes in the loading environment, the sample was divided into two groups (Table 2):

- Pre‐loading group (perinates—≤6 postnatal months): includes infants who are not able to walk independently and rely entirely on their caregivers for transportation (Young & Shapiro, 2018). We expect the lower limbs to be minimally loaded in this group. Children in this phase are not able to fully propel themselves, but start to progressively hold their heads up and sit in an upright position with support.
- Post‐loading group (>6 postnatal months—≤2 years old): includes individuals who have the potential to move more independently. During this period, children start sitting upright without support, they may crawl, and, finally, walk unaided as their gait continues to develop and stabilize (Swan et al., 2020).

**TABLE 2:** Age classes and loading subsets

Age classes	Loading group	Individuals per classes	Total
Perinates/prenatal	Pre‐loading	6	11
0–6 months		5	
6–12 months	Post‐loading	7	17
1.1–2 years		10	

### 2.2 Data 3D acquisition and segmentation

Bones were scanned using different microCT sources (Table 3). Scans were reconstructed as 16‐bit TIFF stacks, and ImageJ v.1.52a (Schneider et al., 2012) was used to inspect the scans and evaluate their quality. When the trabecular bone showed signs of damage or rarefaction due to pathological or diagenetic causes, the specimen was excluded from the analyzes. Avizo v. 9.3 (Thermo Fisher Scientific, Waltham) was used to pre‐process the reconstructed scan data (e.g., crop or resample) and to reconstruct three‐dimensional surface models from the CT data. Where heavy sediment or mummified tissues were present, they were removed in Avizo 9.3 using a Wacom board and the Avizo paint‐brush tool in the label field. A White Hat filter was applied (to the specimens from Norris Farms #36 and some from Velia) to improve the contrast between the bone and the heavy sediment present in the bone. Segmentation of the image data was first performed using the MIA clustering method (Dunmore et al., 2018). In short, this method uses a K‐means algorithm to cluster the CT images into specific classes determined a priori by the user. This clustering is based on voxel or pixel intensity. Therefore, a fuzzy c‐means algorithm (Bezdek et al., 1987; Dunn, 1973) is applied to cope with the scanning artifacts or with different levels of mineralization of the specimen and the soil (Dunmore et al., 2018); then, the class membership probabilities are calculated. Finally, voxels are attributed to the class with the highest membership probability. All the process results in a segmented dataset. All the outputs used here are raw files, linked to MetaImage files (ITK).

**TABLE 3:** Micro‐CT information

Sample	Facility	Type of scanner	Voxel size (μ)
Bologna	Center for Quantitative Imaging (CQI), Pennsylvania State University, PA (USA)	General Electric v|tome|x L300 nano/microCT	20–38
Norris Farms	Center for Quantitative Imaging (CQI), Pennsylvania State University, PA (USA)	General Electric v|tome|x L300 nano/microCT	12–26
Velia	“Abdus Salam” International Centre of Theoretical Physics (ICTP) in Trieste (Italy)	Microfocus X‐ray computed tomography§	18–30
	Rizzoli Institute, Bologna, Italy	Skyscan 1072 system (Bruker Corp., Kontich, Belgium).	18
Perkata	The Abdus Salam International Centre for Theoretical Physics, Trieste, Italy	Microfocus X‐ray computed tomography	18–30

#### 2.2.1 Geometric morphometric analysis

**FIGURE 1:**
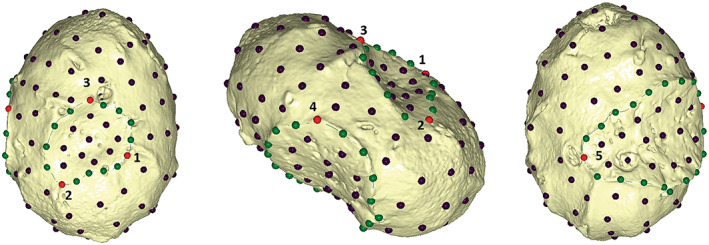
The (semi)landmark configuration. From the left: Dorsal, lateral, and plantar views. Landmarks are shown in red, curve semilandmarks are in green, and surface semilandmarks are represented in purple.

To quantify the morphological differences of the growing talus, a template of 146 (semi)landmarks (five anatomical landmarks, 30 curve semilandmarks, and 111 surface semilandmarks, as shown in Figure 1) was created in Viewbox 4 (dHAL Software) on a perinate talus (Velia T383us2175). This template aims to investigate the morphological changes in the gross morphology of the talus during growth, from the late prenatal weeks to 2 years of age (Figure 1, Table 4). Not every specimen had a fully intact cortical bone, making it impossible to apply the configuration of (semi)landmarks on all. Several tali were therefore excluded from the geometric morphometric (GM) analysis, as reported in Table 4.

**TABLE 4:** Configuration of (semi)landmarks

Description	Type of landmark	*N*.
Landmarks		
1 The most proximo‐medial point of the neck	III	
2 The most proximo‐lateral point of the neck	III	
3 The most anterior point of the neck	III	
4 The most anterior point of the lateral bridge	III	
5 The most medial point of the sulcus tali	III	
Semilandmarks on curves		
Curve L1‐L2: Posterior part of the neck		4
Curve L2‐L3: Lateral and Anterior part of the neck		4
Curve L3‐L1: Medial part of the neck		4
Curve L4‐L5: Posterior margin of the Sulcus Tali		9
Curve L5‐L4: Sulcus tali and bridge		9
Semilandmarks on surface		111

*Note*: Type of landmarks based on (Bookstein, 1997).

The (semi)landmark configurations were applied to all the targets; to minimize the thin‐plate‐spline bending energy (Slice, 2006) between template and targets, and to make them geometrically homologous (Gunz & Mitteroecker, 2013; Mitteroecker et al., 2013) semilandmarks were allowed to slide on curves and surfaces. Coordinates were then registered with a Generalized Procrustes Analysis (GPA) using the R (R Core Team 2020) package geomorph 3.3.1 (Adams & Otárola‐Castillo, 2013). The size was then removed (centroid size, CS = 1); to minimize the Procrustes distance between homologous (semi)landmarks, specimens were translated and rotated. Finally, semilandmarks were allowed to slide against recursive updates of the Procrustes consensus (Rohlf & Slice, 1990; Slice, 2006).

A shape space Principal Components Analysis (PCA) was performed on the Procrustes coordinates to investigate shape modification via the R package Morpho 2.8 (Schlager, 2017). Shapiro Normality Test and Levene test were carried out on the first three principal components (PCs), to evaluate the scattering of the data and its homoscedasticity, respectively. Then, based on the fulfillment of the postulations, the parametric or non‐parametric tests (Analysis of Variance, ANOVA, or Kruskal–Wallis rank‐sum test, respectively) were run to find significant variance between group means along the first three PCs. Pearson's product‐moment correlation was carried out to evaluate if shape variations were related to size (i.e., the natural logarithm of the CS). Procrustes form space was investigated to find any variations of size and shape through PCA by adding the natural logarithm of CS as an additional variable to Procrustes shape coordinates (Klingenberg, 2016; Mitteroecker et al., 2004; Mitteroecker et al., 2013). This procedure reduces shape variation in a few dimensions while preserving size information (Mitteroecker et al., 2004).

#### 2.2.2 Trabecular analyzes

The segmented microCT image data were then analyzed using Medtool 4.3 (Dr. Pahr Ingenieurs e.U, 2017). The trabecular and cortical bone were separated following Gross et al. (2014). Briefly, opening and closing filters of varying kernel sizes (3–5 mm) were applied to the segmented data, followed by a filling procedure designed to algorithmically delineate the interior border of the cortical shell (Pahr & Zysset, 2009). If the excessive cortical porosity of the bone made this procedure difficult, an iterative dilation‐and‐erosion cycle was applied to produce a closed cortical shell. Then, an outer mask (i.e., external surface) and inner mask (i.e., inner surface) are subtracted from the original segmentation to isolate cortical and trabecular bone (Gross et al., 2014). Ultimately, the computational geometry algorithms library CGAL (www.cgal.org) was used to generate a tetrahedral mesh of trabecular bone using the Delauney triangulation (Delaunay, 1934; Gross et al., 2014; Komza & Skinner, 2019).

A 5 mm spherical volume moving along a background grid of 2.5 mm spaced nodes was used to quantify bone volume fraction (BV/TV) and degree of anisotropy (DA) on the segmented volume (Gross et al., 2014; Pahr & Zysset, 2009). The mean intercept length (MIL) approach (Odgaard, 1997), which gave results for first, second, and third eigenvectors and eigenvalues, was used to calculate DA. Then, fabric degree of anisotropy was calculated as (1‐[eigenvalue3/eigenvalue1]) and was scaled between 1 and 0 (1 indicates a highly anisotropic pattern and 0 an isotropic one). The results were interpolated to the centroid of the elements within the tetrahedral meshes, and colormaps were then visualized in Paraview 3.14.1 (Sandia Corporation, Kitware Inc). Mean trabecular thickness (Tb.Th, mm), mean trabecular number (Tb.N), and mean trabecular spacing (Tb.Sp, mm) were also calculated (Hildebrand & Rüegsegger, 1997) and mapped.

In addition, the Phenotypic PointCloud Analysis protocol (DeMars et al., 2021) was used with the BV/TV and DA data to visualize the areas that differ significantly between groups. Due to the differences in the morphology of the developing talus, we used this protocol only for visualization purposes, without making statistical inferences. Trabecular meshes were aligned by employing an adapted version of the MATLAB auto3dgm package (Stephens, 2020), through an algorithm that permits the fully automatic positioning of equivalent points on digital models. The generated pseudolandmarks (i.e., landmark‐like points) can be entered into GMs software (Boyer et al., 2015). A set of 1,200 automatically placed pseudolandmarks was used in this study. Afterward, we performed a GPA using the GeoMorph R package (Adams et al., 2018) to find the mean shape. We then warped the closest‐to‐the‐mean specimen to the mean shape calculated from the GPA (Stephens et al., 2018). This average mesh or canonical specimen was then tetrahedralized with evenly‐spaced (1.75 mm) points using TetWild (Hu et al., 2020), and the vertices of the tetrahedral mesh were converted to a point cloud. Point clouds for each individual were then acquired with the interpolation of BV/TV and DA scalar values to the vertices of the tetrahedral mesh. Next, a python implementation of the Coherent Point Drift algorithm (Myronenko & Song, 2010) was used to align the point clouds by applying the auto3dgm transformation matrices followed by a rigid, affine, and deformable alignment using. BV/TV and DA scalar values were linearly interpolated from each talus' point clouds to the corresponding points in the canonical point cloud using SciPy (Virtanen et al., 2020a, 2020b). The homologous points were compared using a two‐tailed *t*‐test, with *p*‐values corrected for multiple comparisons using random field theory, to control for the chance of false positives (Friston, 1995; Worsley et al., 1996; Worsley et al., 2009), and interactively visualized in Paraview with figures being automatically generated using PyVista (Sullivan & Kaszynski, 2019).

## 3 RESULTS

### 3.1 External morphology

**FIGURE 2:**
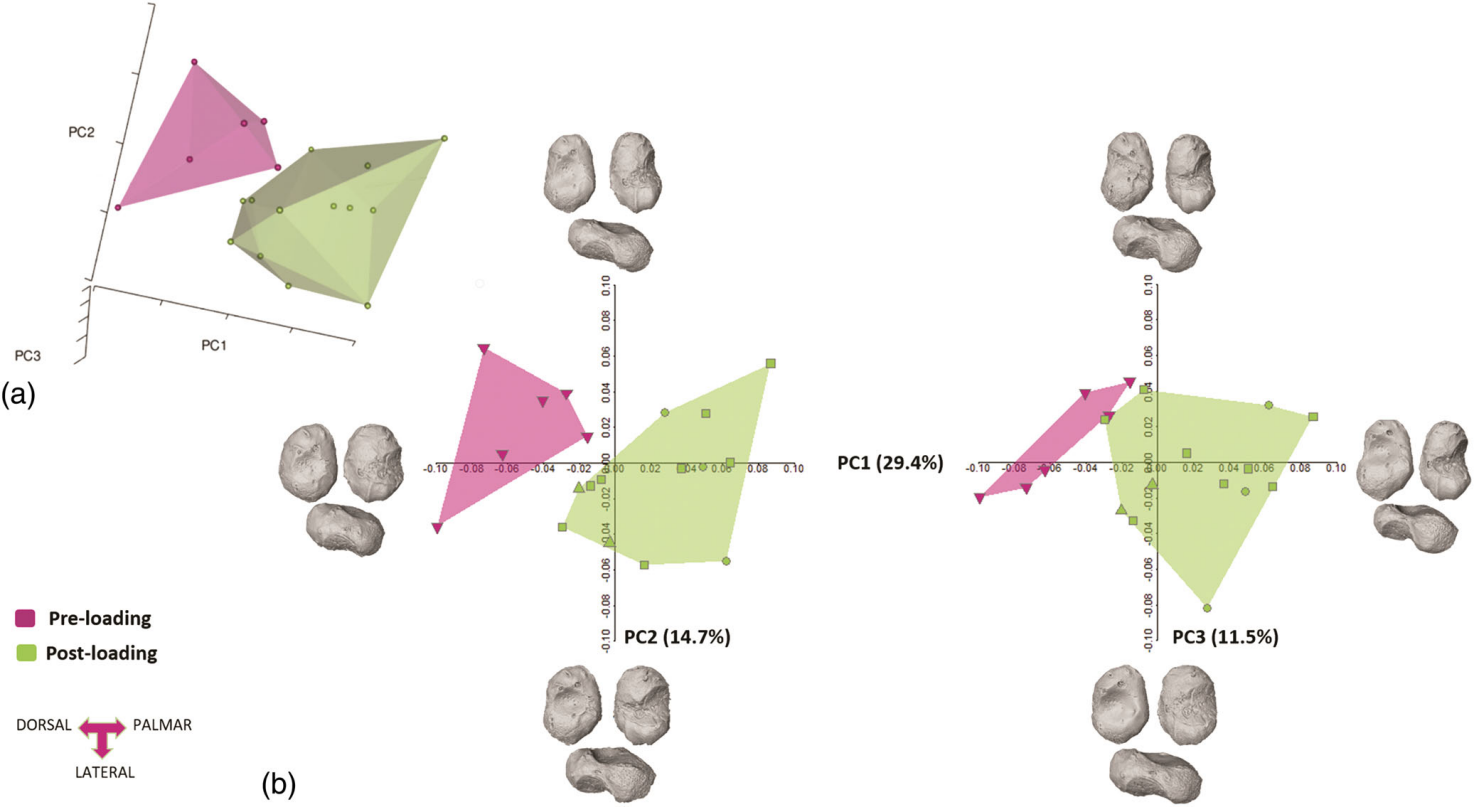
3D shape space plot (a). 2D shape space plot. PC1 and PC2, on the left, PC1 and PC3 on the right. Surface warps of the shape changes along the first three PCs are represented for each group in dorsal, plantar, and lateral views (b). Individuals from Bologna, for which sex was known, are represented by circles (males) and squared symbols (female). Triangles represent the archeological samples.

Results of the first three shape space PCs are shown in Figure 2. Shapiro normality test shows that the first three PCs (55.8% of the total variance) were normally distributed (PC1: *W* = 0.97, *p*‐value = .89; PC2: *W* = 0.96, *p*‐value = .74; PC3: *W* = 0.92, *p*‐value = .13), while the Levene test attested for equality of variance, with *p*‐value > .1 for all the first three PCs.

Most of the morphometric variation is explained by PC1 (29.4%), where the shape scores for PC1 significantly separate the two groups of pre‐loading and post‐loading (ANOVA, *F*‐test 19.6, *p*‐value = .0003). The shape variation described by PC1 is significantly related to size, that is, ontogenetic allometry (*r* = .67, *p*‐value = .001). Negative scores of PC1 account for an overall small, globular morphology of the tali (pre‐loading group), which well represents the not completely ossified neonatal talar morphology (e.g., ossific nuclei), with an already recognizable talar head and neck, as a circular depression, and a well‐defined sulcus tali. The impressions of the cartilage canals are still visible in the posterior part of the body of the talus (Figure 2a,b). Positive scores of PC1 (i.e., post‐loading group) account for a more mature talar body (post‐loading group), with a clear elongation of the entire talus, a longer and wider neck that shows an increase in concavity and surface area, a clear development (though not yet complete) of the trochlea and lateral malleolar facet with the articular facet not yet defined. The area of the sulcus tali is larger and more defined than in the youngest cohort, as is the lateral ridge. The head is slightly rotated medially, and its shape is more similar to the mature talar head. The anterior calcaneal facet is more defined, whereas the posterior calcaneal facet has grown, acquiring a characteristic triangular shape, but is still not well defined.

PC2 scores (14.7%) do not significantly separate the groups (ANOVA, *F*‐test 3.5, *p*‐value = .07), even though they account for a better distinct and deeper sulcus tali and lateral ridge, with a general “slimness” of the shape, that is, less globular in the positive score in respect to the negative ones. The lateral ridge shortens with positive values, due to the growth of the head.

PC3 scores (11.5%) do not show significant differences among groups (ANOVA, *F*‐test 0.003, *p*‐value = .9), although subtle differences can be recognized. Its negative scores show a wider and more concave neck than the one seen in the negative scores of PC1, whereas it becomes narrower and more defined with positive values, which translates into the general elongation of the talar body and the development of the trochlea and head.

In form space, the morphometric variation is explained almost completely by PC1, which accounts for 93.1% of the total variance. The two groups are separated along the *x*‐axis (PC1), for example, strong size difference. Shapiro–Wilk normality test shows that PC scores were normally distributed (*p*‐value > .1), while the Levene test attested for the equality of variance (*p*‐value > .01). ANOVA was performed for PC1 scores, with significant results for both analyzes (*f*‐value = 65.2, *p*‐value = .001). PC2 and PC3 were not significant. As expected, PC1 is strongly correlated with size (*r* = .98, *p*‐value = .001), showing a “growth axis,” while the other PCs are not correlated with size (PC2, *r* = .04, *p*‐value = .8; PC3, *r* = .08, *p*‐value .7). (For additional information, see SOM, Figure S1).

### 3.2 Internal morphology

**FIGURE 3:**
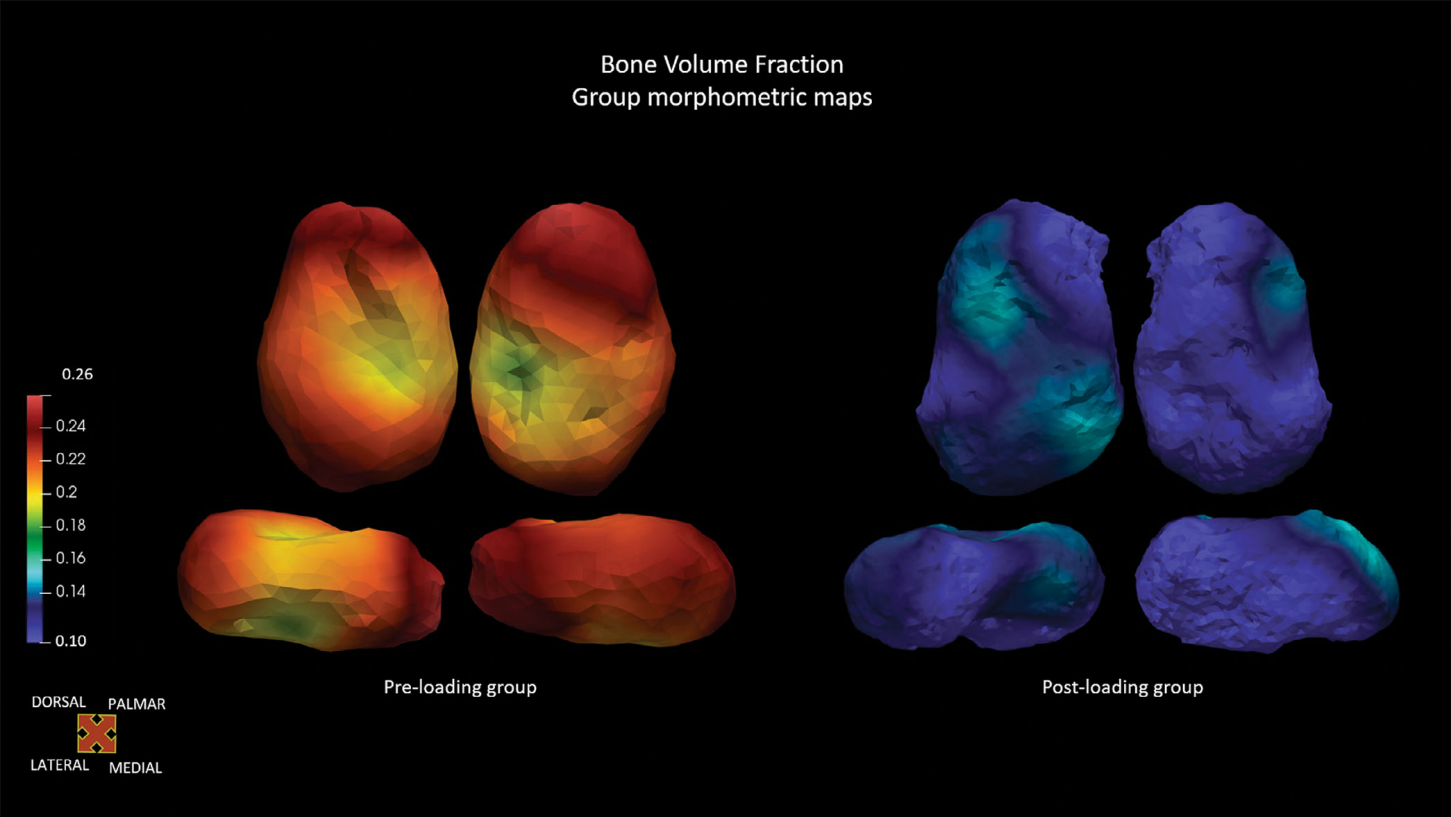
Morphometric maps of the BVTV group means, represented by representative specimens, in dorsal, plantar, lateral, and medial views.

**FIGURE 4:**
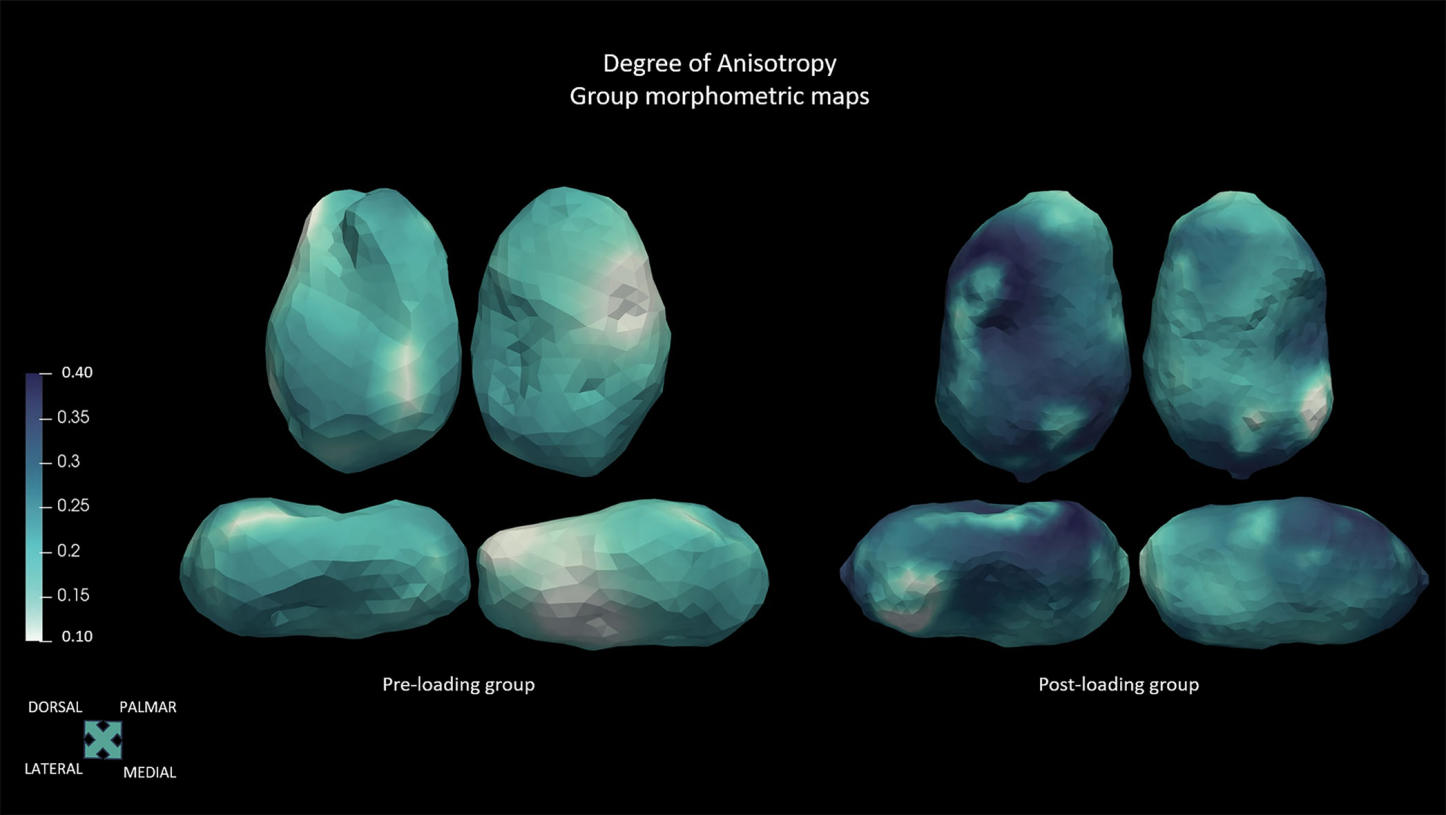
Morphometric maps of the DA group means, represented by representative specimens, in dorsal, plantar, lateral, and medial views.

Trabecular results showed that the architecture in the human talus changes between the pre‐loading and post‐loading groups. Mean, minimum, and maximum values for the two cohorts are listed in Table 5 and shown in Figures 3 and 4. The pre‐loading group shows high values of Tb.N and low Tb.Sp and Tb.Th. In the post‐loading group, Tb.Sp and Tb.Th increase, while Tb.N decreases. BV/TV values are higher in the pre‐loading group, describing a dense trabecular architecture, with the highest values on the medial side of the talus and head. Post‐loading group shows lower BV/TV values, with the highest values in the postero‐lateral part of the trochlea and neck (Figure 3). The architecture is relatively isotropic in the youngest cohort, with the lowest values being on the medial side of the head. Then, DA increases steadily after the initial mechanical load, with the highest values being on the medial side of the talar body, the lateral head, and the medial‐posterior side of the trochlea (Figure 4). Hence, the trabecular architecture changes, from an isotropic, compact, and packed structure with numerous and thin trabeculae in the pre‐loading group, to a more anisotropic and widely‐spaced structure in the post‐loading individuals, with fewer and thicker trabeculae. Individuals' mean values are listed in Tables S1 and S2.

**TABLE 5:** Loading group values

	BV/TV (%)	DA	Tb.N	Tb.Sp	Tb.Th
Pre‐loading	Mean: 21.51	Mean: 0.16	Mean: 1.86	Mean: 0.42	Mean: 0.14
	Min: 9.8	Min: 0.07	Min: 1.1	Min: 0.3	Min: 0.1
	Max: 35.4	Max: 0.26	Max: 2.4	Max: 0.7	Max: 0.14
Post‐loading	Mean: 16.76	Mean: 0.20	Mean: 1.23	Mean: 0.65	Mean: 0.17
	Min: 10.9	Min: 0.16	Min: 0.89	Min: 0.47	Min: 0.13
	Max: 28.4	Max: 0.27	Max: 1.57	Max: 0.92	Max: 0.22

The results of the Phenotypic PointCloud Analysis protocol (Figure S2), for BV/TV and DA, showed, in general, that BV/TV is significantly higher in the dorsal areas of the pre‐loading talus, while DA increases significantly in the medial side of the head and neck of the post‐loading talus.

## 4 DISCUSSION

The main goal of this study was to describe the developmental changes that occurred in the talus from the last prenatal weeks to the end of the first year of life (e.g., ≤2 years), with the aim of better exploring this time frame during which the most important locomotor milestones are hit, and to try to identify bony signals that could be linked to the gradually developing bipedalism. This is, to our knowledge, the first study on the early development of the talus employing both GM and trabecular analysis.

Overall, our results show that the external talar shape separates individuals younger than 6 months of age from those older than 6 months, suggesting that the numerous morphological changes taking place during the first year of life may act on the plastic external shell. This timeframe is pivotal since fundamental milestones are crossed (e.g., sitting upright, crawling, cruising, supported locomotion, and, finally, independent locomotion), and the foot is prepared to support the impending change in mechanical loading. In the pre‐loading group, the talus slowly started changing, with the expansion of the neck surface and an initial and almost imperceptible torsion of the head, ultimately assuming a more elongated shape. This morphology may suggest that during the first 6 months of life, the talus is not ready to bear forces and perform even an immature form of locomotion. The internal morphology reveals a very dense architecture, with numerous and thin trabeculae, which are closely packed, with a rather isotropic structure. During fetal life, the fetus usually punches and kicks against the uterine wall, which represents mechanical resistance (Acquaah et al., 2015). Moreover, hormones play a key role in bone development, with maternal estrogens, in combination with fetal movements, influencing bone development (Bonjour et al., 2012; Gosman et al., 2011; Swan et al., 2020). Furthermore, a “gestational overproduction” with a high bone mass may constitute a postnatal calcium reservoir, helping maintain mineral homeostasis (Acquaah et al., 2015). After birth, this mechanical resistance is lost in the light of the new environment—for example, more space and absence of the uterine wall as a constraint—and the neonatal movements do not produce an increase in load. These results are in line with previous studies, which suggest that a dense and generalized trabecular structure is deposited during endochondral ossification, and that this nonspecialized structure subsequently reorganizes into fewer, thicker, and directionally organized systems of trabeculae (Reissis & Abel, 2012; Ryan & Krovitz, 2006; Ryan & Shaw, 2015; Smith, 2002).

However, in the post‐loading group changes are more noticeable, and pivotal areas (e.g., trochlea, head, and neck) underwent great modifications, suggesting that the foot is preparing to bear the full body weight. The more mature neuromuscular system and external stimuli, for example, caregivers, allow children to start exploring the environment that surrounds them while practicing their first attempts at walking. The foot experiences gradually more loading, even though it is not loaded fully yet. After the 6 months of life, the neck area has expanded considerably, with a slightly more pronounced torsion of the head, a more marked lateral ridge, and an initial expansion of the lateral malleolar process. Talar development is more pronounced, particularly with an increase in size and definition of the articular facets (i.e., trochlea, head, and subtalar calcaneal facets). This timeframe corresponds to when the child experiences more independence and moves more frequently, ultimately engaging in different forms of supported and unsupported locomotion; talar morphology is then subjected to an increase in mechanical load. Though the shape is not mature yet, and far from the adult morphology, during the second year of life (12th–24th month) the locomotion pattern gradually matures. After the rise in loading, the hinted increase in the trochlear curved shape and the expansion of its area suggest that the trochlea may be ready to better receive and distribute the body weight from the tibia. Moreover, the slight increase in the orientation of the head hints that the foot may not yet be performing a full medial weight transfer. This may be consistent with the not‐yet‐developed heel‐strike and toe‐off motion, and medial longitudinal arch. However, Straus (1926) found that a medial torsion of the head was present in the prenatal talus and that it decreases during growth. Our results showed the opposite process. The torsion of the head is pivotal for the correct medial transmission of the forces, though the immature foot does not need to transmit the forces medially yet. As the bodyweight increases, and the gait matures, the torsion of the talar head also increases, aiding this medial weight transfer. This characteristic is thought to be linked to the ontogenetic adduction of the hallux, which is an important phylogenetic signal (Straus, 1926). The development of the posterior calcaneal facet, in particular, plays an important role in distributing the forces while standing and walking. As noted by Hellier and Jeffery (2006), the talar inferior articular facets do not distribute the forces equally while standing: a larger force is transmitted to the navicular rather than to the cuboid (via the calcaneus) and hence, metatarsals are loaded increasingly in a medial direction (Manter, 1946; Salathé et al., 1986). Also, the heel strike gait has not been developed yet, and the child touches the ground with a flat foot. This may explain why the posterior part of the talus, that is, the posterior side of the trochlea and the posterior calcaneal facets, is less developed than the anterior part. However, it is expected to continue developing after 2 years of age, together with the development of a proper heel strike (Zeininger et al., 2018).

Likewise, the internal morphology undergoes multiple changes. BV/TV values decrease, probably as a result of trabecular resorption, with fewer and thicker struts and increased spacing between each other, suggesting that after the onset of bipedal walking, the trabecular architecture changes due to the different loads. Consequently, DA values start to increase slowly. Trabecular architecture models under mechanical loading during gait maturation, as suggested by previous studies (Colombo et al., 2019; Gosman & Ketcham, 2009; Raichlen et al., 2015; Ryan & Krovitz, 2006; Saers et al., 2020). Saers et al. (2020) proposed that excessive bone deposited during endochondral ossification would be resorbed during the first year of life, if insufficiently loaded, following the mechanostat model (Frost, 2003). The decrease in bone volume fraction may be explained by the removal of unloaded or under‐loaded struts (Ryan & Krovitz, 2006). Many studies have found that, while bone volume fraction and trabecular thickness increase, the number of trabeculae decreases during development (Byers et al., 2000; Halloran et al., 2002; Mulder et al., 2005; Nafei et al., 2000; Nuzzo et al., 2003; Parfitt et al., 2000; Raichlen et al., 2015; Ryan & Krovitz, 2006; Saers et al., 2020; Salle et al., 2002; Tanck et al., 2001; Tsegai et al., 2018; Wolschrijn & Weijs, 2004). It appears that initial mechanical adaptation is achieved first by adding bone mass, and then by modeling to gain efficiency. Our results are in line with previous literature and consistent with the BV/TV values that continue to decrease during the first postnatal months as well as with a trabecular structure that is not strained above a certain threshold. This suggests that talar architecture may be able to respond to variations in loading magnitude from the second half of the first year of life.

Recent studies suggest that the initial decrease in BV/TV is not completely driven by mechanical loading, but that it is also programmed and driven by normal developmental processes. Similarly, the decrease in bone mass during the first year of life may be explained as a response to the increased need for calcium during growth (Acquaah et al., 2015), as breast milk contains reduced calcium concentrations due to post‐pregnancy reduction (Ilich & Kerstetter, 2013). According to Land and Schoenau (2008), the decrease in bone mass values they observed in the vertebrae of their samples coincides with elevated parathyroid hormone levels during infancy, which cause an increase in bone resorption and consequent release of calcium. Hence, a developmentally programmed increase in calcium during the prenatal period may serve to create a reservoir for postnatal growth.

The degree of anisotropy progressively increases after six postnatal months. Specifically, values are higher in the trochlea, in the most proximal part of the posterior calcaneal facet, in the neck, and in the superior part of the head, displaying early modeling after strain. This is in accordance with previous literature, which describes the presence of a dense and generalized structure that is initially deposited during endochondral ossification and that later models (Barak, 2020) to cope with the functional role of the talus. This may be linked to early modeling due to the increase in body weight. We did not test for the correlation with body weight, but it is plausible that the weight gain would have played a pivotal role in the morphological changes during early maturation.

Trabeculae are modeled according to the directions of the loading, and as the foot is loaded, struts start to model according to the direction of the forces, that is, compressive and tensile forces in the trochlea and neck areas respectively. Interestingly, DA increases first in the posterior and medial portion of the trochlea, that is, the area which receives the full weight from the tibia. Young walkers, at this stage, touch the ground with flat feet, and the medial arch is not fully developed yet. DA increases also in the area of the neck and lateral side, probably reflecting a more mature locomotion due to the action of the tensile forces in the neck area.

In summary, the external and internal anatomy of the human talus provides insight into the development of pedal loading in humans. The external morphology shows us how the foot, for example, talus, might have been loaded during its different growth stages: first reflecting the absence of gait, then reflecting the increase in loading. With the increase in weight, maturation of the neuromuscular system, and biological growth of children, the talar morphology commences showing the readiness to walk, first by increasing the trochlear surface, then by starting to develop the subtalar joints and changing the orientation of the head. However, further analyzes are needed to better understand if, and to what extent, the morphological modifications previously described are signals related to the development of bipedal gait. The study of non‐human tali or the comparison with tali from individuals who have never walked due to pathological conditions could shed some light on the morphological changes most closely related to the development of bipedalism.

The internal architecture provides precious information about how the bone was actually loaded, and it tells us that the immature morphology of the shell well corresponds to the immature and not loaded trabecular architecture. Interestingly, BV/TV is higher in the trochlea, in the lateral process, and in the subtalar facets, which have a great increase in growth after the first year. Trabecular changes may correspond to the ontogenetic changes that we explore on the external shell of the talus. Also, these changes may be linked to the initial development of motor skills in juveniles.

## 5 CONCLUSION

This investigation shows how the talar morphology changes in concert with the onset of bipedal locomotion. This study confirms our initial hypotheses, demonstrating that in the early stages of growth the talus responds by adapting to mechanical strain. This work fills the existing gap in the literature, exploring how the trabecular bone in the juvenile talus starts modeling during early stages, showing once again the fast response of the trabecular bone to mechanical stimuli.

Unfortunately, a few limitations need to be highlighted. First of all, the relatively small sample size limits the analyzes, which is further limited in the cortical analysis due to the often unpreserved cortical bone, for example, post‐depositional damages. Moreover, the youngest ages are even less represented. We are aware of the fact that fetal and perinatal individuals are rare to find, but these categories need deeper investigation. Second, locomotor and cultural behaviors of these individuals are not known, and the unbalanced populations here represented may mask slight interpopulation differences, for example, cultural and genetic. Future works should focus on the childrearing practices to better understand the locomotor behaviors of the populations under study.

Despite these drawbacks, this study demonstrates that the pre‐walking phase is clearly reflected in talar morphology, both in the internal and external morphologies. With an increased sample, this contribution may help identify differences in human ontogenetic trajectories, and better explore the dynamics that guide the early talar development, that is, how much is the contribution of the genetic blueprint in this phase. The morphologies of pre‐ and post‐locomotion are well‐differentiated and may help in distinguishing these two fundamental phases.

This is the first time that the early talar changes are explored in such a holistic way. We have shown also that the exploration of both external and internal morphologies offers a valuable key to the interpretation of plastic changes. Ultimately, our findings may help in shedding light on the early development of the human foot and could be a useful instrument in the study of the juvenile fossil record.

## AUTHOR CONTRIBUTIONS

**Carla Figus:** Conceptualization (lead); data curation (lead); formal analysis (equal); investigation (lead); validation (equal); visualization (equal); writing – original draft (lead); writing – review and editing (lead). **Nicholas B. Stephens:** Data curation (supporting); formal analysis (equal); software (lead); supervision (supporting); validation (equal); visualization (equal); writing – review and editing (supporting). **Rita Sorrentino:** Data curation (supporting); formal analysis (supporting); writing – review and editing (supporting). **Eugenio Bortolini:** Formal analysis (supporting); validation (supporting); writing – review and editing (supporting). **Simona Arrighi:** Writing – review and editing (supporting). **Owen A. Higgins:** Writing – review and editing (supporting). **Federico Lugli:** Writing – review and editing (supporting). **Giulia Marciani:** Writing – review and editing (supporting). **Gregorio Oxilia:** Writing – review and editing (supporting). **Matteo Romandini:** Writing – review and editing (supporting). **Sara Silvestrini:** Writing – review and editing (supporting). **Fabio Baruffaldi:** Resources (supporting); writing – review and editing (supporting). **Maria Giovanna Belcastro:** Resources (supporting); writing – review and editing (supporting). **Federico Bernardini:** Resources (supporting); writing – review and editing (supporting). **Anna Festa:** Resources (supporting). **Tamás Hajdu:** Resources (supporting); writing – review and editing (supporting). **Orsolya Mateovics‐László:** Resources (supporting); writing – review and editing (supporting). **Ildiko Pap:** Resources (supporting); writing – review and editing (supporting). **Tamás Szeniczey:** Writing – review and editing (supporting). **Claudio Tuniz:** Resources (supporting); writing – review and editing (supporting). **Timothy M. Ryan:** Conceptualization (supporting); resources (supporting); supervision (equal); validation (equal); writing – review and editing (supporting). **Stefano Benazzi:** Conceptualization (supporting); funding acquisition (lead); project administration (lead); resources (lead); supervision (equal); validation (equal); writing – review and editing (supporting).

## Supporting information


**Appendix S1** Supporting Information

## Data Availability

The data that support the findings of this study are available from the corresponding author upon reasonable request.
