# Supplementary material for: Morphologies in‐between: The impact of the first steps on the human talus
Source: Anat Rec (Hoboken). 2022 Jun 21;306(1):124–42. doi: 10.1002/ar.25010 (PMC10083965; doi:10.1002/ar.25010)
Supplement: Supplementary file 1 — Appendix S1 Supporting Information [file AR-306-124-s001.docx]

**Supplementary information**

**Morphologies in-between: the impact of the first steps on the human talus**

Carla Figus^1*^, Nicholas B Stephens^2^, Rita Sorrentino^3,1^, Eugenio Bortolini^1,4^, Simona Arrighi^1^, Owen A Higgins^1^, Federico Lugli^1^, Giulia Marciani^1,5^, Gregorio Oxilia^1^, Matteo Romandini^1^, Sara Silvestrini^1^, Fabio Baruffaldi^6^, Maria Giovanna Belcastro^3^, Federico Bernardini^7,8^, Anna Festa^6^, Tamás Hajdu^9^, Orsolya Mateovics-László^10^, Ildiko Pap^9,11,12^, Tamás Szeniczey^9^, Claudio Tuniz^8,13^, Timothy M Ryan^2^, Stefano Benazzi^1,14^


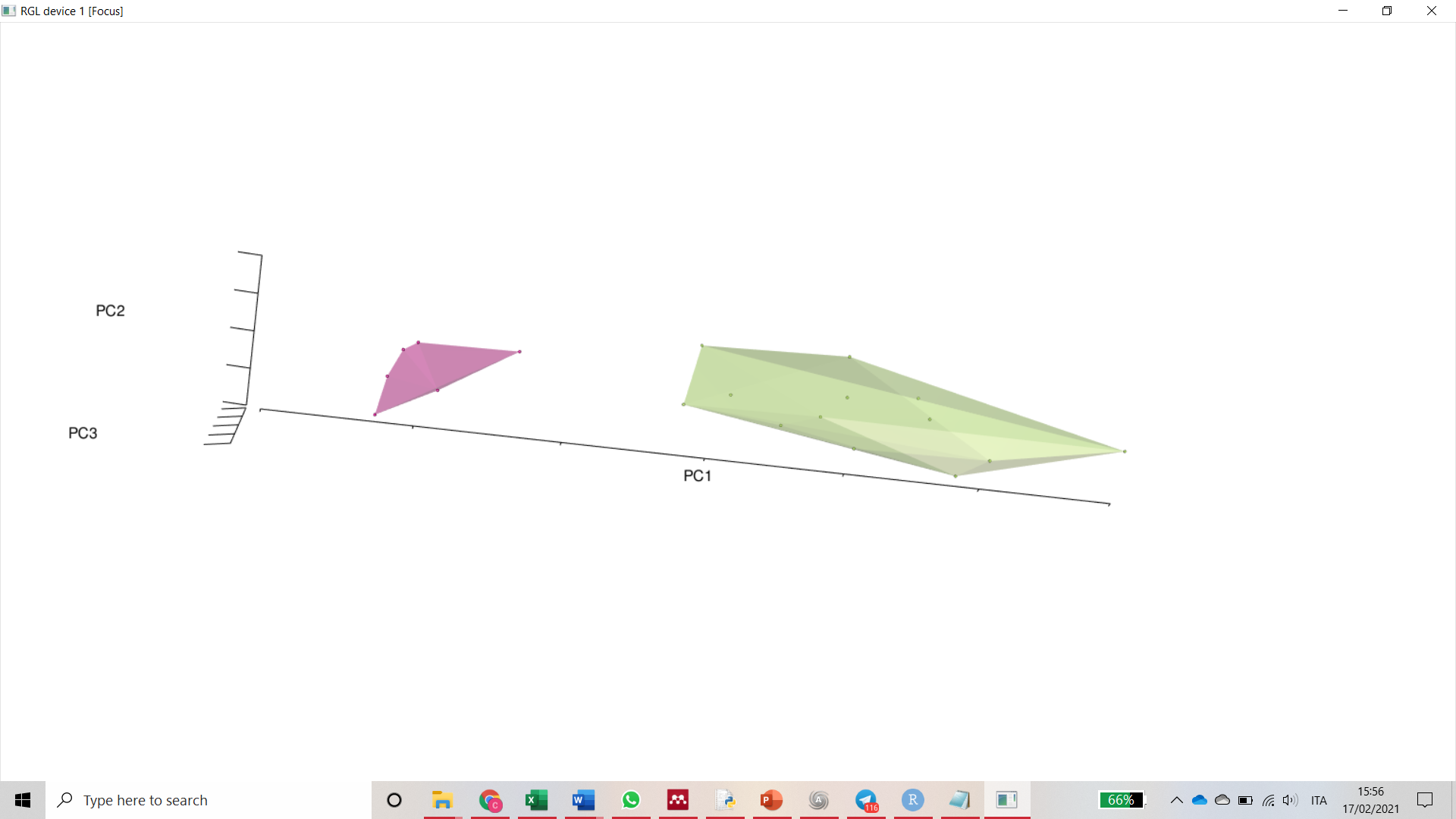


Figure S1 - 3D form space plot with loading groups. The two groups are well separated in form space.

Table S1 – Individuals’ averages for Tb.N, Tb.Sp, Tb.Th

| **Name** | **Age Class** | **Loading group** | **Age at death** | **Tb.N mean** | **Tb.Sp mean (SD)** | **Tb.Th mean (SD)** |
| --- | --- | --- | --- | --- | --- | --- |
| VeliaT322. | Perinates | Pre-load | 36-39 weeks | 1.72 | 0.4 (0.22) | 0.14 (0.04) |
| VeliaT315. |  | Pre-load | 36-40 weeks | 1.87 | 0.37 (0.15) | 0.15 (0.04) |
| VeliaT344. |  | Pre-load | perinates | 2.09 | 0.31 (0.13) | 0.16 (0.05) |
| VeliaT350. |  | Pre-load | perinates | 1.53 | 0.5155 (0.2455) | 0.13 (0.04) |
| VeliaT383. |  | Pre-load | 38 weeks ca | 1.88 | 0.40 (0.17) | 0.12 (0.02) |
| VeliaT417. |  | Pre-load | perinates | 2.41 | 0.26 (0.12) | 0.14 (0.03) |
| VeliaT305. | 0-6 postnatal  months | Pre-load | 0-3 months | 1.98 | 0.37 (0.21) | 0.12 (0.02) |
| NF821369. |  | Pre-load | 8 weeks | 1.72 | 0.47 (0.20) | 0.10 (0.02) |
| NF821045. |  | Pre-load | 0-6 months | 2.21 | 0.32 (0.17) | 0.12 (0.04) |
| VeliaT300. |  | Pre-load | 0-6 months | 1.14 | 0.73 (0.36) | 0.13 (0.03) |
| VeliaT441. |  | Pre-load | 0-6 months | 1.86 | 0.40 (0.21) | 0.13 (0.02) |
| NF821051. | 6.1-12 months | Post-load | 7.5 months | 1.11 | 0.68 (0.32) | 0.21 (0.06) |
| VeliaT442. |  | Post-load | 6-9 months | 1.57 | 0.49 (0.23) | 0.14 (0.03) |
| VeliaT368. |  | Post-load | 0.75-1 year | 1.55 | 0.51 (0.23) | 0.13 (0.02) |
| VeliaT289. |  | Post-load | 9-12 months | 1.36 | 0.57 (0.25) | 0.15 (0.03) |
| BO58_M. |  | Post-load | 11 months | 1.53 | 0.47 (0.18) | 0.17 (0.04) |
| BO60_F. |  | Post-load | 11 months | 1.37 | 0.58 (0.26) | 0.14 (0.03) |
| NF820614. |  | Post-load | 12 months | 1.05 | 0.81 (0.29) | 0.13 (0.02) |
| VeliaT415. | 1.1-3 years | Post-load | 1-1.5 years | 1.41 | 0.53 (0.22) | 0.17 (0.04) |
| VeliaT434. |  | Post-load | 1-1.5 years | 1.27 | 0.58 (0.20) | 0.20 (0.04) |
| BO14_M. |  | Post-load | 1 years and 5 months | 1.21 | 0.68 (0.24) | 0.13 (0.03) |
| NF821014. |  | Post-load | 1.5 years | 1.19 | 0.58 (0.24) | 0.25 (0.06) |
| NF821046. |  | Post-load | 1.5 years | 1.14 | 0.68 (0.33) | 0.18 (0.04) |
| PerkataNyuli655. |  | Post-load | 1-3 years | 0.89 | 0.92 (0.50) | 0.19 (0.03) |
| VeliaT286. |  | Post-load | 1.5-2 years | 1.22 | 0.63 (0.32) | 0.18 (0.04) |
| BO14_F. |  | Post-load | 1.9 years | 1.30 | 0.61 (0.22) | 0.15 (0.04) |
| NF821026. |  | Post-load | 2 years | 0.90 | 0.92 (0.52) | 0.18 (0.04) |
| NF821207. |  | Post-load | 2 years | 0.94 | 0.86 (0.35) | 0.18 (0.05) |
| PerkataNyuli516. |  | Post-load | 1.5-3 years | 1.07 | 0.70 (0.26) | 0.22 (0.07) |

Table S2 – Individuals’ averages for BV/TV and DA

| **Name** | **Age Class** | **Loading group** | **Age at death** | **BV/TV (%)** | **Degree of Anisotropy** |
| --- | --- | --- | --- | --- | --- |
| VeliaT322. | Perinates | Pre-load | 36-39 weeks | 21.49 | 0.09 |
| VeliaT315. |  | Pre-load | 36-40 weeks | 26.54 | 0.18 |
| VeliaT344. |  | Pre-load | Perinate | 29.14 | 0.13 |
| VeliaT350. |  | Pre-load | Perinate | 14.14 | 0.31 |
| VeliaT383. |  | Pre-load | 38 weeks ca | 20.41 | 0.13 |
| VeliaT417. |  | Pre-load | Perinate | 35.44 | 0.07 |
| VeliaT305. | 0-6 months | Pre-load | 0-3 months | 21.65 | 0.16 |
| NF821369. |  | Pre-load | 8 weeks | 9.82 | 0.12 |
| NF821045. |  | Pre-load | 0-6 months | 23.68 | 0.11 |
| VeliaT300. |  | Pre-load | 0-6 months | 12.41 | 0.26 |
| VeliaT441. |  | Pre-load | 0-6 months | 21.92 | 0.17 |
| NF821051. | 6.1-12 months | Post-load | 7.5 months | 18.53 | 0.16 |
| VeliaT442. |  | Post-load | 6-9 months | 18.12 | 0.19 |
| VeliaT368. |  | Post-load | 0.75-1 year | 18.95 | 0.17 |
| VeliaT289. |  | Post-load | 9-12 months | 18.47 | 0.16 |
| BO58_M. |  | Post-load | 11 months | 23.24 | 0.16 |
| BO60_F. |  | Post-load | 11 months | 20.12 | 0.26 |
| NF820614. |  | Post-load | 12 months | 5.70 | 0.24 |
| VeliaT415. | 1.1-2 years | Post-load | 1-1.5 years | 20.66 | 0.13 |
| VeliaT434. |  | Post-load | 1-1.5 years | 18.6 | 0.27 |
| BO14_M. |  | Post-load | 1 years and 5 months | 11.8 | 0.23 |
| NF821014. |  | Post-load | 1.5 years | 28.42 | 0.17 |
| NF821046. |  | Post-load | 1.5 years | 14.84 | 0.20 |
| PerkataNyuli655. |  | Post-load | 1-3 years | 10.93 | 0.24 |
| VeliaT286. |  | Post-load | 1.5-2 years | 21.31 | 0.16 |
| BO14_F. |  | Post-load | 1 years and 9 months | 13.82 | 0.26 |
| NF821026. |  | Post-load | 2 years | 10.58 | 0.20 |
| NF821207. |  | Post-load | 2 years | 10.51 | 0.21 |
| PerkataNyuli516. |  | Post-load | 1.5-3 years | 17.18 | 0.21 |

**
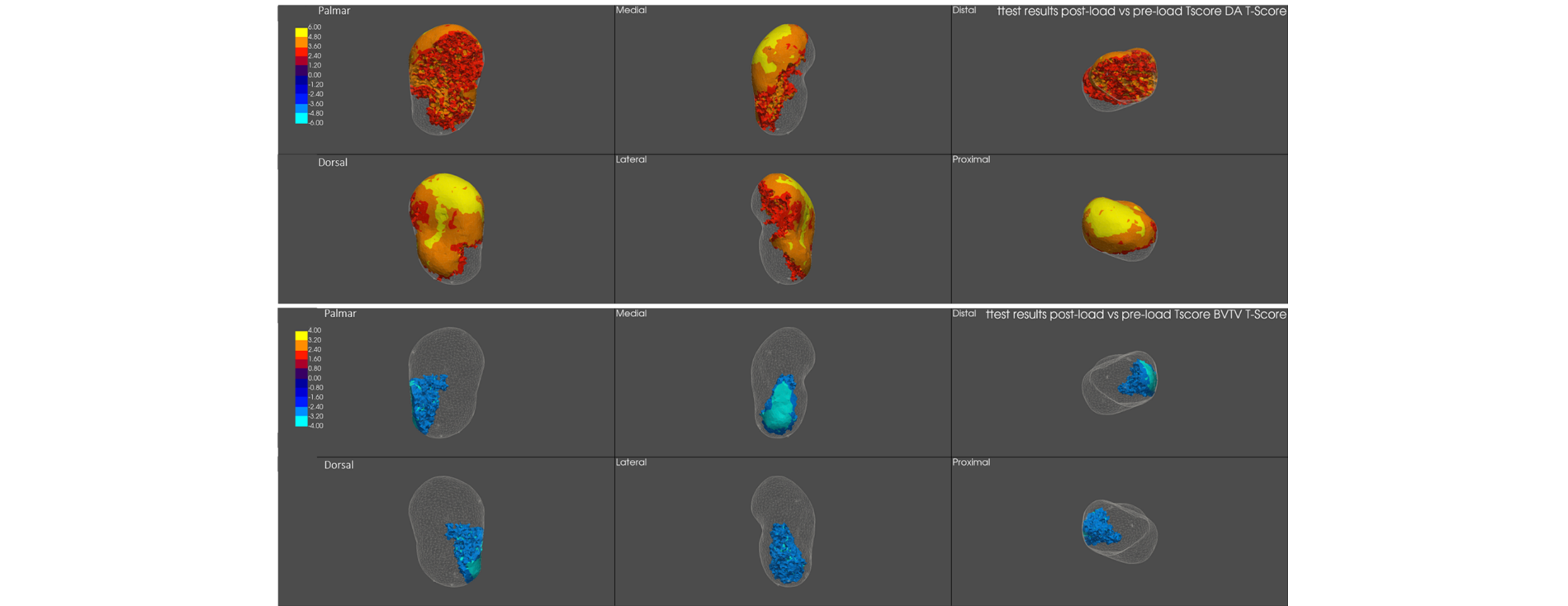
**

*Figure S2 –* *Warm colors represent the pre-loading group, cold colors the post-loading group.*

*In the upper two rows: BV/TV t-test results showing the significantly different areas between the two groups.*

*In the lower two rows: DA t-test results showing the significantly different areas between the two groups.*
